# Genetic Tests for Ecological and Allopatric Speciation in Anoles on an Island Archipelago

**DOI:** 10.1371/journal.pgen.1000929

**Published:** 2010-04-29

**Authors:** Roger S. Thorpe, Yann Surget-Groba, Helena Johansson

**Affiliations:** School of Biological Sciences, Bangor University, Bangor, United Kingdom; University of Arizona, United States of America

## Abstract

From Darwin's study of the Galapagos and Wallace's study of Indonesia, islands have played an important role in evolutionary investigations, and radiations within archipelagos are readily interpreted as supporting the conventional view of allopatric speciation. Even during the ongoing paradigm shift towards other modes of speciation, island radiations, such as the Lesser Antillean anoles, are thought to exemplify this process. Geological and molecular phylogenetic evidence show that, in this archipelago, Martinique anoles provide several examples of secondary contact of island species. Four precursor island species, with up to 8 mybp divergence, met when their islands coalesced to form the current island of Martinique. Moreover, adjacent anole populations also show marked adaptation to distinct habitat zonation, allowing both allopatric and ecological speciation to be tested in this system. We take advantage of this opportunity of replicated island coalescence and independent ecological adaptation to carry out an extensive population genetic study of hypervariable neutral nuclear markers to show that even after these very substantial periods of spatial isolation these putative allospecies show less reproductive isolation than conspecific populations in adjacent habitats in all three cases of subsequent island coalescence. The degree of genetic interchange shows that while there is always a significant genetic signature of past allopatry, and this may be quite strong if the selection regime allows, there is no case of complete allopatric speciation, in spite of the strong *primae facie* case for it. Importantly there is greater genetic isolation across the xeric/rainforest ecotone than is associated with any secondary contact. This rejects the development of reproductive isolation in allopatric divergence, but supports the potential for ecological speciation, even though full speciation has not been achieved in this case. It also explains the paucity of anole species in the Lesser Antilles compared to the Greater Antilles.

## Author Summary

Over the last 150 years, since Darwin's study of islands and his “Origin of Species,” island archipelagos have played a central role in the understanding of evolution and how species multiply (speciation). Islands epitomise the conventional view of geographic (allopatric) speciation, where genomes diverge in isolation until accumulated differences result in reproductive isolation and the capacity to coexist without interbreeding. Current-day Martinique in the Lesser Antilles is composed of several ancient islands that have only recently coalesced into a single entity. The molecular phylogeny and geology show that these ancient islands have had their own tree lizard (anole) species for a very long time, about six to eight million years. Now they have met, we can genetically test for reproductive isolation. However, when we use selectively neutral markers from the nuclear genome, on this naturally replicated system, we can see that these anoles are freely exchanging genes and not behaving as species. Indeed, there is more genetic isolation between adjacent populations of the same species from different habitats than between separate putative allospecies from the ancient islands. This rejects allopatric speciation in a case study from a system thought to exemplify it, and suggests the potential importance of ecological speciation.

## Introduction

Speciation generates biodiversity and is therefore a key process in evolution and ecology, and the relative importance of factors contributing to speciation in sexually reproducing animals, such as genetic drift in spatial isolation, natural selection, sexual selection and mutation-order, remains an active area of research [1]–[8]. Since neo-Darwinism [9] the most conventional view of speciation in sexually reproducing animals has been by the accumulation of differences by genetic drift and selection in allopatry. While there has been growing paradigm shift towards models [10] and processes such as ecological speciation [1], [5], [7], [8] that are not dependent on allopatry, there have been few critical tests of allopatric speciation in systems which are regarded as exemplifying the process, such as island archipelagos [9], [11]–[17]. This is primarily because, from a contemporary perspective, genetic isolation cannot be assessed in spatially isolated populations. However, a historical perspective allows us to test the genetic isolation of anole species isolated for a very substantial time before their islands coalesced.

**Figure 1:**
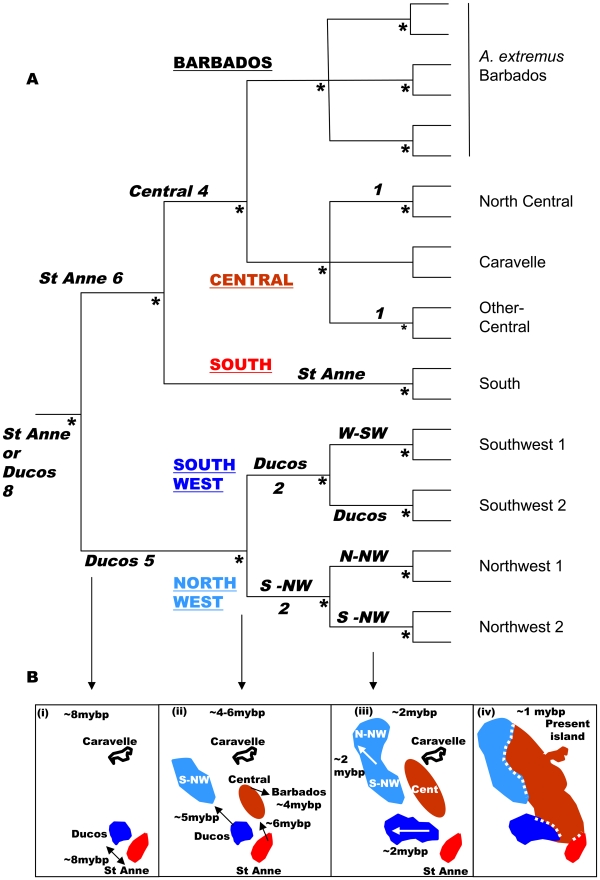
Phylogeny and geohistory. (A) Summmarized cladogram based on Cytochrome *b* Bayesian gene tree [21]. The five main lineages are Barbados and the four precursor island lineages, south, central, northwest and southwest (underlined upper case), the suggested locality and time of origin (to the nearest million years before present) are in bold italics with the times based on geological calibration [21], which gives values close to those based on a general lizard clock. Terminal nodes (light font) are representative minor lineages of *Anolis roquet* from Martinique, and *A. extremus* from Barbados. Nodes with a posterior probability of 1.00 are marked with an asterisk. (B) Summarized historical scenario (dates as above) for the Martinique and Barbados regions and their *Anolis roquet/extremus* lineages. The first phylogenetic division at circa 8 mybp (at, or just before the origin of the recent island arc) saw the establishment of Ducos (southwest) and the St Anne peninsular (south) as separate populations (B i), then the central region was colonized from St Anne at circa 6 mybp and the northwest precursor island from the recent arc was colonized from Ducos (southwest) at circa 5 mybp (B ii). Barbados (*Anolis extremus*) was colonized from the central region soon after (circa 4 mybp) (B ii). Finally, with the uplift of the central region (B iii, iv) the northwest, southwest and southern precursor islands were joined into the single island of Martinique at circa 1 mybp (B iv).

**Figure 2:**
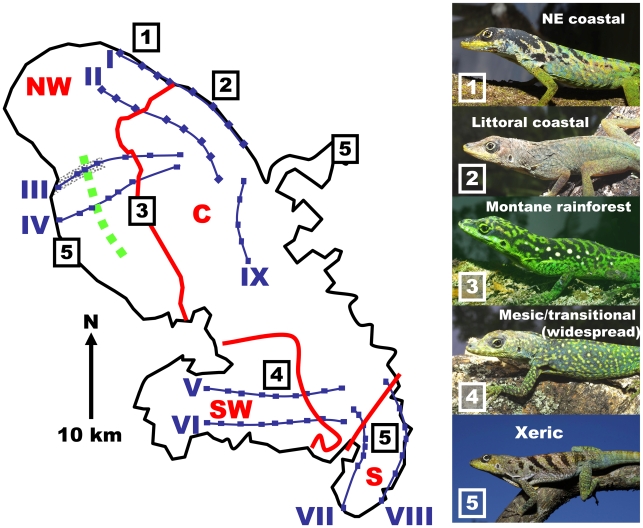
Precursor island regions, lineages, transects, and ecotone on Martinique. Geological boundaries between precursor islands are indicated by red lines. They are occupied by lineages labelled in red (NW = northwest, C = central, SW = southwest, S = south). The ecotone between xeric coastal and montane rainforest is indicated by a green, broken line, and the section of Transect III devastated by the 1902 pyroclastic surges that destroyed St Pierre [31] is indicated by grey shading. Transects and their sites are in blue and numbered with blue Roman numerals I to IX. The control transect (IX) within the central mesic zone, is without an ecotone or secondary contact. The photographs are of adult male anoles (locality on Martinique indicated by boxed numbers), 1 northeastern coastal, 2 littoral coastal form, 3 montane rainforest form, 4 widespread mesic/transitional form, and 5 xeric form occurring in the western rainshadow, St Anne Peninsular in the south and the eastern tip of the Caravelle peninsular in east where annual rainfall is circa 1500mm a year or less [50].

*Anolis* (small insectivorous lizards) is the most speciose amniote genus (circa 400 species) [18] and show little inter-specific hybridization [19]. Just two colonizations of the Caribbean islands have resulted in 150 species, so they may be thought of as exemplifying allopatric speciation in island archipelagos [11]–[14], [18], [20]. These anole radiations appear to have inhabited the Lesser Antilles since the origin of the younger island arc, or just before (i.e circa 8–9 mybp) with a southern and a northern series [21]. On what is currently recognized as the island of Martinique (southern series), the paraphyletic anole *Anolis roquet* has deep phylogeographic divisions, with *Anolis extremus* from Barbados nested within it [21]. Geology [22]–[23], molecular phylogeography and molecular clock analysis [21] reveals that four precursor islands of Martinique (Figure 1) are associated with four mtDNA lineages of ‘*A. roquet*’. The island ages, molecular clock and geographic distribution of the lineages link closely to suggest that the precursor islands of Martinique (together with Barbados) had separate anole allospecies for up to about 8mybp, before central uplifting joined the Martinique precursors to form a single island (with Barbados remaining independent) [21]. This gave three secondary contact zones in Martinique (Figure 2) between previously allopatric forms (south-central, SW-central, NW-central) that:- 1) are phylogenetically deeper than the species-level split between *A. extremus* (consistently regarded as a valid species [18]) and its sister clade within *A. roquet* [21]; 2) have diverged a substantial time ago (6–8 mybp) and have a level/time of phylogenetic divergence that is comparable to other Lesser Antillean anole species [11]–[12]; 3) may show distinct mtDNA lineages with almost no haplotype inter-digitation [21]; and 4) may show a *prima facie* case for parapatric bimodality in multivariate quantitative traits at some points of contact [24] (Figure S1). Hence, this is an appropriate test of allopatric speciation.

Martinique anoles also provide a test for ecological speciation, or isolation by adaptation [7]. The quantitative traits of Lesser Antillean anoles adapt by natural selection to environmental zonation, as shown by common garden and natural selection experiments [25]–[26], parallels among island species, and correlation studies that take phylogenetic history into account [27]. In Martinique, the montane rainforest and coastal xeric woodland are distinctly different habitats with pronounced differences in the environmental conditions across the ecotone between them. As with other Lesser Antillean anoles, the Martinique anole adapts to these conditions and their populations show marked habitat-related differences in quantitative traits such as morphology (shape, color, pattern and scalation) and dewlap hue [21],[24], resulting in distinct ecotypes. The ecotone between these coastal xeric and montane rainforest habitats provides a test for ecological speciation for comparison with secondary contact zones.

Hence, with the Martinique anole there is the potential for speciation to occur in accordance with both an allopatric model (where the different lineages on precursor islands speciate), and an ecological model (where the different ecotypes speciate). Preliminary analysis of a single transect suggested that under specific circumstances there may be greater restriction of genetic exchange between habitat types than previously allopatric forms in secondary contact [21]. However, this analysis examined just one of the three pairs of coalescing islands (northwest *vs.* central), under only one set of selection regimes (strong convergent selection for montane rainforest on both lineages where they met along that transect). Furthermore, this study was not replicated and no control was used, limiting the capacity to generalize, and raising several questions. Specifically, do the other pairs of coalescing precursor islands populations (southwest-central and south-central) show evidence of genetic isolation or not; is the pattern of inter-digitation of the mtDNA lineages and introgression of the neutral nDNA consistent along the length of each of the three secondary contact zones, or does it vary dependant on other factors; how do the selection regimes along the transect influence the extent of genetic isolation among previously allopatric forms; under what ecological conditions (extent and abruptness of habitat change) is there restricted genetic exchange among habitat types and how long does it take to develop?

To answer these questions we investigated the xeric/rainforest ecotone and all three cases of island coalescence, each with two to four replicate transects, together with a control transect (Figure 2, Table 1, Table 2). By measuring nuclear genetic structure, mtDNA lineage, quantitative traits and climate variation along these replicated transects, across both geological and habitat contact zones, we are able to critically test the role of these two factors, and their interaction, in the differentiation of island anoles. We show that, although there is always a signature of past allopatry in the nuclear genetic structure, and this can be quite strong dependent on the comparative selection regimes across the secondary contact zone, there is no complete allopatric speciation for any of the three allopatric pairs. Instead, if there is sufficient magnitude and abruptness of habitat change, then there is even greater differentiation across the ecotone, and this can develop over a brief period of time. Although the ecological speciation is not complete, it has reached what has been characterized as a “later stage” in the speciation continuum [7]. This supports a relatively important role for ecological speciation under the appropriate circumstances [1], [5], [7], [8].

**Table 1:** Transect attributes: geology, lineage, and traits.

Transect	Precursor island lineages	Number of sites	Geology lineage fit^a^			Correlation QTs v lineage^b^		Allopatric speciation predicted
			ϕ	*P*	n	r	*P*	
I	Northwest/Central	8	0.95	<0 .001	384	0.97	0.001	Y
II	Northwest/Central	8	0.80	<0 .001	381	0.89	0.003	Y
III	Northwest/Central	7	0.72	<0 .001	236	0.47	0.282	Y
IV	Northwest/Central	7	0.90	<0 .001	313	0.39	0.388	Y
V	Southwest/Central	9	0.77	<0 .001	420	0.73	0.026	Y
VI	Southwest/Central	9	0.84	<0 .001	420	0.29	0.450	Y
VII	South/Central	8	0.75	<0 .001	326	0.93	0.001	Y
VIII	South/Central	7	0.61	<0 .001	307	0.61	0.145	Y
IX	Central. Control	5	-	-	-	-	-	N

a Goodness of fit (ϕ) between geological precursor island and mtDNA lineage frequency across all individuals from each site (n).

b Correlations between site mean CV scores for QTs and lineage frequency (n = number of sites).

**Table 2:** Transect attributes: climate, habitat, and traits.

Transect	Precursor island lineages	Habitat along transect	Variation in climate^a^	Variation in QTs^b^	Correlation QTs v climate^c^		Ecological speciation predicted
					r	*P*	
I	NW/Central	coastal	0.15	10.6	0.94	<0.001	N
II	NW/Central	transitional	0.08	8.5	0.79	0.021	N
III	NW/Central	coast to montane	2.25	15.8	0.97	<0.001	Y
IV	NW/Central	coast to montane	2.69	16.9	0.96	<0.001	Y^3^
V	SW/Central	mesic^1^	1.12	4.5	0.69	0.040	N
VI	SW/Central	mesic	1.48	5.3	0.36	0.341	N
VII	South/Central	xeric^2^	0.67	6.4	0.81	0.014	N
VIII	South/Central	xeric	0.30	4.4	0.88	0.009	N
IX	Central. Control	mesic	1.05	6.9	0.43	0.470	N

a Magnitude of variation (site maximum-site minimum) along transect for climate in principal component scores.

b Magnitude of variation along transect for quantitative traits (QTs) in canonical variate scores normalized to unit within-group standard deviation.

c Correlations between site mean CV scores for QTs and climate scores (n = number of sites).

1 There is an altitudinal gradient along transect V.

2 There is a transition to a more mesic habitat in the north of this transect.

3 See the text for a caveat to this prediction.

## Results/Discussion

### Geological island precursors and mtDNA lineages

Recent island-wide phylogenetic studies identified four main mtDNA lineages within *A. roquet* whose geographical limits correspond very closely to the geological junctions between precursor islands [21], [28], with the timing of divergence between these lineages compatible with the age of the different precursor islands. This supports the scenario illustrated in Figure 1B, which suggests that the individual lineages evolved in allopatry for about 6mybp (central-south) to 8 mybp (central southwest and central northwest) until the precursor islands merged to form present day Martinique. Here, we use a large sample per site, with sites along transects focussed on the contact zones. Estimating the frequency of mtDNA lineages at localities along these transects enables us to test for any inter-digitation of the lineages and the fit between the distribution of the lineages and the precursor islands at this fine scale.

**Figure 3:**
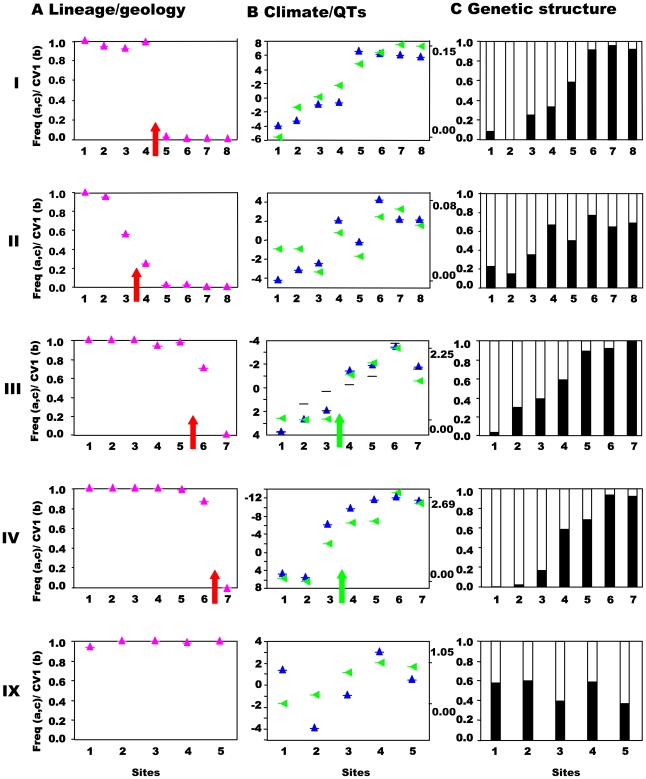
Northwest-central precursor islands: lineages, quantitative traits, and genetic structure along transects. Transects (I–IV) across the secondary contact between the putative allospecies from the northwest and central precursor island and ecotones (III, IV), together with control transect (IX). Sites 1-n are along the horizontal axes. (A) Lineage and geology. Mitochondrial DNA lineage frequencies are indicated as mauve triangles, n≈48 per site), with the red arrow indicating the geological boundary between precursor islands. (B) Climate and Quantitative Traits. Mean canonical variate scores for quantitative traits are indicated by blue upright triangles (left axes in units of within-group standard deviations) for sites based on combined morphology (e.g., scalation, proportions, pattern) and dewlap hue characters. Principal component score of climate data (green triangles) scaled from zero (right axis, max and min values). The green arrow indicates the position of an ecotone. In transect III dewlap hue CV scores are represented by blue triangles and heteroscedastic morphological traits by black horizontal bars. (C) Genetic structure. Frequency of individuals assigned by Bayesian cluster analysis based on variation in nine hypervariable, neutral nuclear microsatellites where the number of clusters is set to two for comparative purposes (see Table 3).

**Figure 4:**
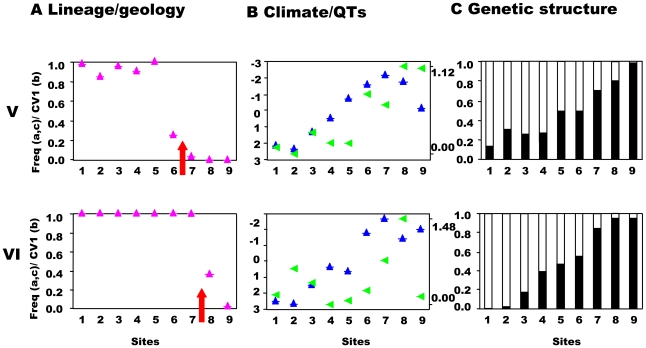
Southwest-central precursor islands: lineages, quantitative traits, and genetic structure along transects. Transects (V,VI) across the secondary contact between the putative allospecies from the southwest and central precursor island. See Figure 3 for control transect (IX) and legend.

**Figure 5:**
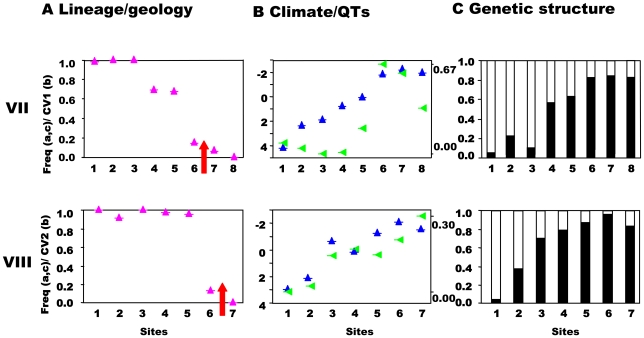
South-central precursor islands: lineages, quantitative traits, and genetic structure along transects. Transects (VII,VIII) across the secondary contact between the putative allospecies from the south and central precursor island. See Figure 3 for control transect (IX) and legend.

With the exception of transect VIII (ϕ = 0.61), we observed a very close association between the precursor islands and the mtDNA lineages (0.71<ϕ<0.95) and little, or almost no (transects I,IV), inter-digitation, even at this fine spatial scale (Table 1, Figure 3, Figure 4, Figure 5). This absence of substantial inter-digitation, despite a relatively long period of contact (the precursor islands merged about 1 Mya [21]), implies the absence of extensive female-driven gene flow [29] between these previously allopatric lineages.

### Habitat and quantitative trait variation

Climate is a strong determinant of habitat and can be objectively measured and quantified. The results (Table 2) show strong climatic variation along the transects (III, IV) that run from the xeric coast to the montane rainforest with a sharp transition (ecotone) between these habitats (Figure 3). Other transects generally run within habitat types and show more subtle climatic variation, i.e., are without abrupt changes in habitat of a high magnitude.

A wide-ranging multivariate profile of the quantitative traits (QTs) of individuals was taken (these include both spectrometric dewlap hues [21] and morphological traits such as colour pattern, body dimensions and scalation) to estimate the change in QTs along a transect in relation to habitat type, ecotone and lineage. Lesser Antillean anole quantitative traits (QTs) are generally tightly linked to the habitat and have generally been shown to adapt to environmental conditions and reflect selection regime rather than phylogeographic lineage [24]–[28]. Hence, as predicted, the large magnitude of habitat variation in transects III and IV is matched by a high magnitude of QT variation (16–17 within group standard deviations, Table 2) with highly divergent rainforest and xeric ecotypes (Figure 2 and Figure 3), and a close correlation between QTs and climate variation along the transect (r = 0.96 to 0.97, Table 2). The large magnitude and close association of the climate and QT variation indicates the potential importance of the ecotone in determining population structure. Elsewhere, where the magnitude of climatic variation along a transect is less (because they largely run within habitat types), such a very high correlation between climate and QTs is not predicted or observed. Even so, the correlation is only insignificant in one non-control transect (Table 2), once again suggesting the general importance of habitat type in determining quantitative traits.

The correlation between quantitative traits and lineage frequency is significant along four transects (Table 1), and is particularly high in transect I, where, on this spatial scale, there is no overlap between multivariate morphology of morphs either side on the lineage contact zone (Figure S1).

### Predictions and tests of allopatric speciation

The allopatric model of speciation predicts that the four lineages that spent a substantial time in isolation on separate islands (divergence at circa 6–8my) should all be reproductively isolated entities. That is, there should be four species with very little (if any) gene exchange among them where they meet along all three contact zones (northwestern/central, southwestern/central and southern/central) on what is currently Martinique. The very close association (with only one exception) between the precursor islands and the mtDNA lineages along the transects does not contradict this (Table 1).

This prediction was tested by estimating the population structure along transects I–VIII using neutral, hypervariable, nuclear microsatellite markers, primarily analysed by Bayesian assignment, with support from AMOVA and standardised F_ST_′ values. Principal component analysis (PCA) provides an independent perspective on the population affinities. The analysis of these markers along the replicated transects across these three zones clearly rejects the presence of reproductively isolated (or even partially isolated) species. This is the case for all three precursor island contacts: the central/northwest contact (Figure 3), the central/southwest contact (Figure 4), and the central/south contact (Figure 5) along the length of the contact for all replicates and various types of selection regimes (but see transect I below). The Bayesian assignment method detects two clusters in most transects (Table 3, Table S1), but the transition between the two clusters generally is not closely associated with the lineages and/or forms a smooth cline (Figure 3, Figure 4, Figure 5). The PCA (Figure S2) supports the Bayesian clusters in transects I–VIII as the pattern of relative frequency of the Bayesian clusters (where K = 2) is almost identical to the pattern of PC1 scores for each transect (r = 1.0 with one exception).

**Table 3:** Nuclear genetic structure.

Transect	Clusters^a^	Fit to allopatric Speciation^b^			Fit to ecological Speciation^c^		
	K	ϕ	*P*	n	ϕ	*P*	n
I	2	0.68	<0 .001	383			
II	1	0.40	<0 .001	380			
III	2	0.33	<0 .001	327	0.62^1^	<0 .001	327
IV	2	0.37	<0 .001	325	0.71	<0 .001	325
V	2	0.45	<0 .001	414			
VI	2	0.51	<0 .001	421			
VII	2	0.39	<0 .001	376			
VIII	2	0.32	<0 .001	332			
IX	1	-			-		

Bayesian Assignment.

a Recognised number of Bayesian clusters (K) in neutral hypervariable nuclear markers (microsatellites), see methods.

b Goodness of fit (ϕ) between Bayesian assignment (K = 2, or assumed to be 2 for comparison) based on microsatellites and categories predicted by allopatric speciation across n individuals along transect.

c Goodness of fit (ϕ) between Bayesian assignment (K = 2, or assumed to be 2 for comparison) based on microsatellites and categories predicted by ecological speciation across n individuals along transect.

1 See the text for a caveat to this prediction.

The association between nuclear genetic clusters and allopatric speciation model (lineage categories) is, with one exception, modest (0.32<ϕ<0.51) even if significant (Table 3) and ϕ does not approach unity (complete isolation). This pattern is supported by the AMOVA and standardized F_ST_′ values. The AMOVA show sporadic significant structure associated with lineages (transects I, II, V, VII), but all the Φ_CT_ values are substantially less than unity and too low for reproductive isolation (Table 4). Similarly, mean standardized genetic differentiation between pairs of populations on each side of lineage boundaries is low to moderate (0.072<F_ST_′<0.166, Table 4). This suggests high levels of nuclear gene exchange between lineages (the equivalent unstandardized F_ST_ values are 0.014<F_st_<0.043). Of particular interest are transects III and IV where the nuclear genetic structure associated with the northwest and central lineages can be compared directly with that associated with habitats (Table 3 and Table 4). Here the Bayesian clusters show substantially poorer fit to the allopatric speciation model (0.32<ϕ<0.37) than the habitat categories (0.62<ϕ<0.71). The AMOVA shows low (−0.00064<Φ_CT_<0.00158) and insignificant Φ_CT_ for the lineage categories, but higher (0.03481<Φ_CT_<0.01665) and significant Φ_CT_ for the habitat categories. The mean standardized genetic differentiation is also much lower between lineage categories (0.072<F_ST_′<0.075) than habitat categories (0.137<F_ST_′<0.213).

**Table 4:** Nuclear genetic structure.

Transect	AMOVA^a^				F_ST_′^b^	
	Structure by Lineage		Structure by Habitat		Lineage	Habitat
	Φ_CT_	*P*	Φ_CT_	*P*	F_ST_′	F_ST_′
I	0.00774	0.03128			0.146	
II	0.00874	0.01955			0.106	
III	0.00158	0.28250	0.01665	0.02737	0.075^1^	0.137^2^
IV	−0.00064	0.59433	0.03481	0.02835	0.072^1^	0.213^2^
V	0.00633	0.01955			0.098	
VI	0.01282	0.05572			0.137	
VII	0.01209	0.02737			0.120	
VIII	0.01096	0.09286			0.166	
IX					0.087^3^	

AMOVA and F_ST_′.

a AMOVA was used to test groups of localities along each transect divided by lineage, and where appropriate (transects III, IV) also by habitat.

b Mean of pairwise standardized F_ST_′ values across the lineage contact zone (I–VIII) or ecotone (III,IV).

1 Values computed between lineages, within habitat (rainforest).

2 Values computed between habitats, within lineage (northwest lineage).

3 Values computed between each pair of localities in this control transect for comparison.

In general, while there may be a nuclear genetic signature of past allopatry for all four mtDNA lineages associated with precursor islands, there is no allopatric speciation. The partial exception to this general trend is transect I, where the central and northwest lineages meet on the northeast coast. Here, there is almost no inter-digitation of mtDNA lineage markers (ϕ = 0.95), and a sharp stepped cline in quantitative traits at the junction of the precursor islands (Figure 3B). There is some genetic isolation between the lineages as shown by the Bayesian assignment (ϕ = 0.68, Figure 3C), although neither AMOVA nor standardized F_ST_′ values are exceptionally high (Table 4). If these lineages were equally isolated along their entire secondary contact zone there might have been a rather weak case for partial allopatric speciation and recognition of their status as separate species. However, they are not. Even along the adjacent transect (II) in the transitional forest, which is only 5km inland, the lineages show little genetic isolation (no Bayesian clusters, Table 3, Table S1, Figure 3C) and do not have distinct quantitative traits (Figure 3B). Further along this secondary contact zone in the montane rainforest (transects III, IV) the quantitative traits are identical either side of the secondary contact zone with little nuclear genetic isolation estimated from Bayesian assignment, AMOVA or standardized F_ST_′. Direct experimental measures of selection in Lesser Antillean [25], [26], and other [18], [30] anoles, as well as other studies of adaptation [21], [24], [27], have shown strong selection intensity on anole quantitative traits, and the pattern of climate variation and QT variation along transects differs among transects I–IV. Hence, although the populations from transects I–IV may broadly share the same history (particularly adjacent transects I and II), they differ in the pattern and intensity of selection along the transect. The similarity of the environment either side of this secondary contact zone in the rainforest (transects III, IV, Figure 3B), and the remarkably parallel appearance of these northwestern and central lineages forms in the rainforest [28] (Figure 2, image 3) suggests strong convergent selection working on these populations. Along the coastal transect (I) there may be no such strong convergent selection, and indeed the environmental variables show a smooth cline along the transect so there may be some divergent selection. This suggests that the persistence of a strong genetic signal of past allopatry may be contingent on the pattern of selection regimes.

In conclusion, even though there has been a substantial period of allopatric divergence between northwest/central (8 mybp), southwest/central (8 mybp) and south/central (6 mybp) lineages, and only restricted inter-digitation of the mtDNA, there is no evidence of complete allopatric speciation even though there may be a significant signal of past allopatry. This is consistent across all three pairs of putative allospecies and between the replicates along the length of all three contact zones, irrespective of the pattern of selection regimes. Nevertheless, if the pattern of selection allows, a stronger signal of past allopatry may be retained. Overall, the results are compatible with divergence in allopatry followed by substantial introgression on secondary contact due to a lack of reproductive isolation.

### Predictions and tests of ecological speciation

The distinctly different habitats of the xeric coast and the montane rainforest, associated with strongly divergent quantitative traits, provide an opportunity to test for ecological speciation along transects III and IV (Table 2). Bayesian assignment indicates that both transects have restricted genetic exchanges across the xeric-montane ecotone, although this is stronger in transect IV (ϕ = 0.71) than III (ϕ = 0.62). The populations of *Anolis* in the area of transect III (Figure 2) were most likely severely impacted by the 1902 pyroclastic surge that destroyed St Pierre [31]. Although the reinstatement of the reduced gene exchange associated with the ecotone may have been facilitated by ecotypes colonizing the vacant area from adjacent populations of the same altitude, anoles can readily colonize adjacent areas of different attitudes. Consequently, perhaps to some extent, the signal of restricted genetic exchange may have to have developed in circa 100 years, which is likely to be much shorter than elsewhere along this ecotone, and may be too short even for ecological differentiation in these terrestrial amniotes [32].

The results of the AMOVA also support a reduction of gene exchange between habitats for the two transects. This test shows a significant structure when the sites are grouped according to their habitat, but not when they are grouped according to their lineage (Table 4, Table S1). Similarly, the mean standardized genetic divergence (F_ST_′) is much higher between habitats than lineages (see above). Even if this is not full reproductive isolation, the restriction of gene exchange between the habitats is very substantial, and along transect IV it is greater than any in this study. Moreover, (with the above caveat regarding altitudinal restrictions on re-colonization) it may be capable of developing rapidly as transect III shows greater isolation than associated with allopatric divergence with the AMOVA and (with one exception) the goodness of fit (ϕ) statistics.

Nosil *et al* [7] recognise several stages in the continuum of ecological speciation: 1) population differentiation, 2) ecotype formation, 3) speciation and 4) post-speciation divergence. They suggest that increased genotypic clustering (as evidenced here) indicates a later stage of the speciation process, and the degree of genetic isolation here is as great, or greater, than that associated with their [7] example of the most reproductively isolated *Pundamilia* cichlid pairs. Moreover, the adjacent, and environmentally very comparable, island of Dominica also has distinct anole ecotypes. A study of microsatellite variation among anole populations on Dominica did not indicate genetic clustering of the ecotypes [27], so Martinique anoles appear to be at a later stage than the stage 2 of the Dominican ecotypes. Hence, although it is clear that this there is no full ecological speciation here, it appears that the Martinique anoles are between the ecotype (2) and speciation (3) stages in the ecological speciation continuum. It may be that the situation is in equilibrium, or is a stage in a progression towards greater isolation. Moreover, even if progression to greater isolation was possible, it could be prevented by persistent volcanic disturbance of the ecotone and/or its spatial discontinuity.

Both natural and sexual selection may play a role in this ecological pattern of gene exchange as predation pressure for crypsis [33] may interact with the need for conspecific communication. Substantial work on Lesser Antillean anole ecotypes, including natural selection experiments, indicates that a wide range of character systems, rather than just single characters, adapt these ecotypes to the specific biotope [24]–[27]. Hence, natural selection will be impacting many independent traits [7]. Moreover, sensory drive may be important [34] as these habitats have different light conditions which may impact on visual conspecific communication via secondary sexual traits, including dewlap hue. If assortative mating occurs, where a female preferentially chooses a male with the appropriate pattern and hue for that habitat, then this could result in reduced gene exchange among populations in different habitats.

### Implications

This replicated population genetic study robustly and consistently suggests that, across a range of opportunities and conditions, there is pronounced introgression after allopatry and that even a very substantial amount of time in spatial isolation does not, on its own, necessarily allow for the development of reproductive isolation and speciation. This is all the more notable as fertile, natural inter-specific hybrids are extremely rare in this large, well-studied, genus [18], [19], and this is a radiation that is generally regarded as exemplifying allopatric speciation [11]–[14], [18], [20]. Even though the habitat forms are partially, rather than completely, reproductively isolated, they can show greater isolation than the putative allospecies, and it may be that this can develop rapidly. In addition, the extent of the genetic signature of past allopatry may be dependent on the pattern of selection regimes across the secondary contact. These observations have implications for animal speciation in general and speciation in anoles in particular. While one could choose to emphasize the lack of complete ecological speciation in this case, we believe these observations reveal the potential importance of ecological divergence as a contributory factor in speciation, including in situations where ecological divergence initiates speciation, but does not complete it [7], and where allopatry is important, but adaptation to environmental differences are also required, as recently suggested for speciation in birds [35]. Consequently, a role for ecology in speciation, including ecological speciation, or isolation by adaptation [1], [5], [7]–[8], [32], [36]–[38], may be of widespread relevance, and non-allopatric models [10] should not be excluded from consideration. These implications are particularly relevant to the most speciose amniote genus, *Anolis*, including the large Greater Antillean communities, where sympatric and parapatric speciation have been regarded as not being an important phenomena in anole evolutionary diversification [14], [18], [20]. Finally, it contributes to an explanation of why there are so few species of *Anolis* in the Lesser Antilles compared to the Greater Antilles [14]. At the stage of the allopatric model where species number on an island is increased by colonization from other islands [14], the colonizers interbreed with the species already on the island, because no reproductive isolation has developed while they are in allopatry. The genetic signal of this interbreeding is then lost because the number of overseas colonizers per unit time will be vanishingly small compare to the turnover in the large endemic population.

## Methods

### Samples

Replicate transects were taken across each precursor island junction (Figure 2); northwest lineage to central lineage transects I, II, III and IV, southwest lineage to central lineage transects V and VI, south to central lineage transects VII and VIII, with a control transect (IX) within the central lineage. The number of sites per transect was 8, 8, 7, 7, 9, 9, 8, 7 and 5 respectively for transects I to IX. At each site 48 naturally autotomized tail-tip biopsies were sampled for molecular analysis, while quantitative traits and dewlap hue were recorded from ten adult males. Where transects crossed the same lineages and were in broadly comparable habitats (eg, III+IV, V+VI, VII+VIII) samples were collected, and data was recorded and analysed in these transect pairs.

### Lineages

The lineages were first investigated using complete cytochrome *b* sequence from the mtDNA. PCR-RFLP analyses were then designed to efficiently assign numerous individuals to a specific lineage (northwest, southwest, south or central). The cyt *b* fragment used in the phylogeographic analysis was digested after amplification using the restriction enzyme SspI (New England Biolabs) for 3 hours at 37°. The digested products were run on a 2% agarose gel containing ethidium bromide. This enzyme distinguishes between the central lineage (uncut by this enzyme), the southern lineage (cut at position 598) and the clade comprising the southwestern and the northwestern lineages (cut at position 166). To further distinguish between southwest and northwest lineages, we digested the same fragment using the restriction enzyme DraI (New England Biolabs) that cuts the PCR products from the northwest lineage at position 227, while those from SW lineage were uncut by this enzyme.

### Climate

The habitat type at each site was estimated from a multivariate climatic profile using nineteen climatic variables from Worldclim (http://www.worldclim.org/). These variables were annual mean temperature, mean diurnal range, isothermality, temperature seasonality, maximum temperature warmest month, minimum temperature coldest month, temperature annual range, mean temperature wettest quarter, mean temperature driest quarter, mean temperature warmest quarter, mean temperature coldest quarter, annual precipitation, precipitation wettest month, precipitation driest month, precipitation seasonality, precipitation wettest quarter, precipitation driest quarter, precipitation warmest quarter, and precipitation coldest quarter. Logarithm (natural) transformed data was subjected to principal component analysis. The component defining the climatic trend along the transect was plotted and the magnitude of climatic change in this trend can be taken as the range between maximum and minimum component scores. If there was an ecotone the cut-point between habitat types was defined as the midpoint between these maximum/minimum component scores.

### Quantitative traits

A multivariate suite of 21 morphological characters (colour pattern, trunk hue, scalation, body dimensions) were recorded [21], [39]. The hue of the anterior and posterior dewlap was recorded using reflectance spectrometry [21] and the spectrum of each was divided into 6 independent hues following a multiple-group eigenvector procedure [21], [40]. The morphological and spectrometric characters were then subjected to canonical analysis with the CVs scaled so that the pooled within-group standard deviation was unity. Heteroscedasticity was a problem with transect III so, as an alternative, a principal component analysis was also run on normalized site means for this transect.

### Genetic structure (neutral nDNA)

The samples were genotyped at nine nuclear microsatellite loci (AAE-P2F9, ABO-P4A9, AEX-P1H11, ALU-MS06, ARO-035, ARO-062, ARO-065, ARO-120, ARO-HJ2) [41]–[43] in a single multiplex using a Qiagen Multiplex PCR kit with the annealing temperature at 55°. PCR products were then analysed on an ABI 3130xl genetic analyser and the genotypes scored using Genemapper v4.0 (Applied Biosystems). Hardy-Weinberg equilibrium and linkage disequilibrium were tested for using Genepop v3.4 [44]. After Bonferoni correction, there were no consistent departures from Hardy-Weinberg equilibrium, or linkage disequilibrium. Only one locus in one population showed a significant departure from Hardy-Weinberg equilibrium (transect I, site 8 for locus ARO-HJ2), and there was only one significant association between loci ALU-MS06 and ARO-035 in one population (transect IV site 3).

The primary genetic structure along each transect was studied using Bayesian clustering performed by the program STRUCTURE v2.1 [45]. We defined the number of populations (K) from 1 to 9 and 10 independent runs were performed for each value of K using the admixture model, a burn-in of 100,000 steps followed by 400,000 post burn-in iterations. We determined the optimal number of populations using the maximum value of the posterior probability of the data [45]. We also used AMOVA, performed by Arlequin v3.11 [46], to test for genetic differentiation predicted by alternative speciation models. Within each transect populations were grouped by modal lineage, or, where appropriate, by habitat. For two transects (III, IV), where both types of speciation could have occurred, this allowed direct comparison of competing speciation hypotheses. Finally, the mean genetic differentiation among populations either side of a lineage, or habitat, boundary along a transect was estimated by calculating the mean standardized pairwise F_ST_′ using RecodeData v0.1 [47] and FSTAT v2.9.3 [48]. To give an independent perspective on the population affinities revealed by the Bayesian clustering we performed principal component analysis (PCA) of transect site gene frequencies using PCAGEN [49]. For each transect the PC1 site scores were compared to Bayesian site frequencies (where K = 2) by correlation.

### Goodness of fit and correlation tests

The relationship between lineage, genetic isolation, past allopatry, ecotone, climate, and adaptive quantitative traits was investigated at sites along a series of replicated transects (Figure 2) across the secondary contact zones (transects I to VII) and ecotone (transects III and IV). Transect IX did not cross any lineage boundary or ecotone and was used as a control transect.

#### Geology, lineage, and quantitative traits

For each transect, we estimated the goodness of fit, phi (ϕ), between the geological precursor islands and the mtDNA lineage frequency, where ϕ was calculated as √ (χ^2^/n), and χ^2^ was based on a 2×2 contingency table. For each transect, we also calculated correlation between the site mean CV scores for QTs and the mtDNA lineage frequency at each site.

#### Climate, habitat, and quantitative traits

For each transect, we calculated the correlation between the site mean CV scores for QTs and the site PC scores for climatic variables, as well as estimating the magnitude of variation in QTs (highest site mean CV minus lowest site mean) and climate (highest site PC score minus lowest site score).

#### Genetic clusters and tests of speciation

Individuals were assigned to a genetic cluster based on Bayesian assignment with K = 2, and to a category predicted by allopatric speciation based on the modal mtDNA lineage of the site. As a test of allopatric speciation, goodness of fit (ϕ) was then calculated between genetic clusters and predicted allopatric species via a χ^2^ based on a 2×2 contingency table (for complete speciation ϕ approaches unity). Similarly, individuals were assigned to a category predicted by ecological speciation based on the habitat type of the site. As a test of ecological speciation, goodness of fit (ϕ) was then calculated between genetic clusters and predicted ecological species via a χ^2^ based on a 2×2 contingency table.

## Supporting Information

Figure S1 Bimodality in quantitative traits. Frequency histogram of individuals along coastal transect I showing bimodality in quantitative traits at this spatial scale. The variable is canonical variate 1 (units in within-group standard deviations). Northwestern precursor individuals are the right mode, central precursor individuals are the left mode, without any overlap.
(0.02 MB PDF)

Figure S2 Principal component analyses. Principal component 1 score (vertical axis) for each site (horizontal axis) for transect I = VIII. The legend is as for Figure 3, except r is the correlation between the PC1 score and the frequency of Bayesian clusters when K = 2.
(0.03 MB PDF)

Table S1 Mean posterior probabilities (over 10 replicates) for STRUCTURE for K clusters. Maximum values are in bold.
(0.01 MB PDF)
