# Supplementary material for: Genetic Tests for Ecological and Allopatric Speciation in Anoles on an Island Archipelago
Source: PLoS Genet. 2010 Apr 29;6(4):e1000929. doi: 10.1371/journal.pgen.1000929 (PMC2861690; doi:10.1371/journal.pgen.1000929)
Supplement: Figure S1 — Bimodality in quantitative traits. Frequency histogram of individuals along coastal transect I showing bimodality in quantitative traits at this spatial scale. The variable is canonical variate 1 (units in within-group standard deviations). Northwestern precursor individuals are the right mode, central precursor individuals are the left mode, without any overlap. (0.02 MB PDF) [file pgen.1000929.s001.pdf]

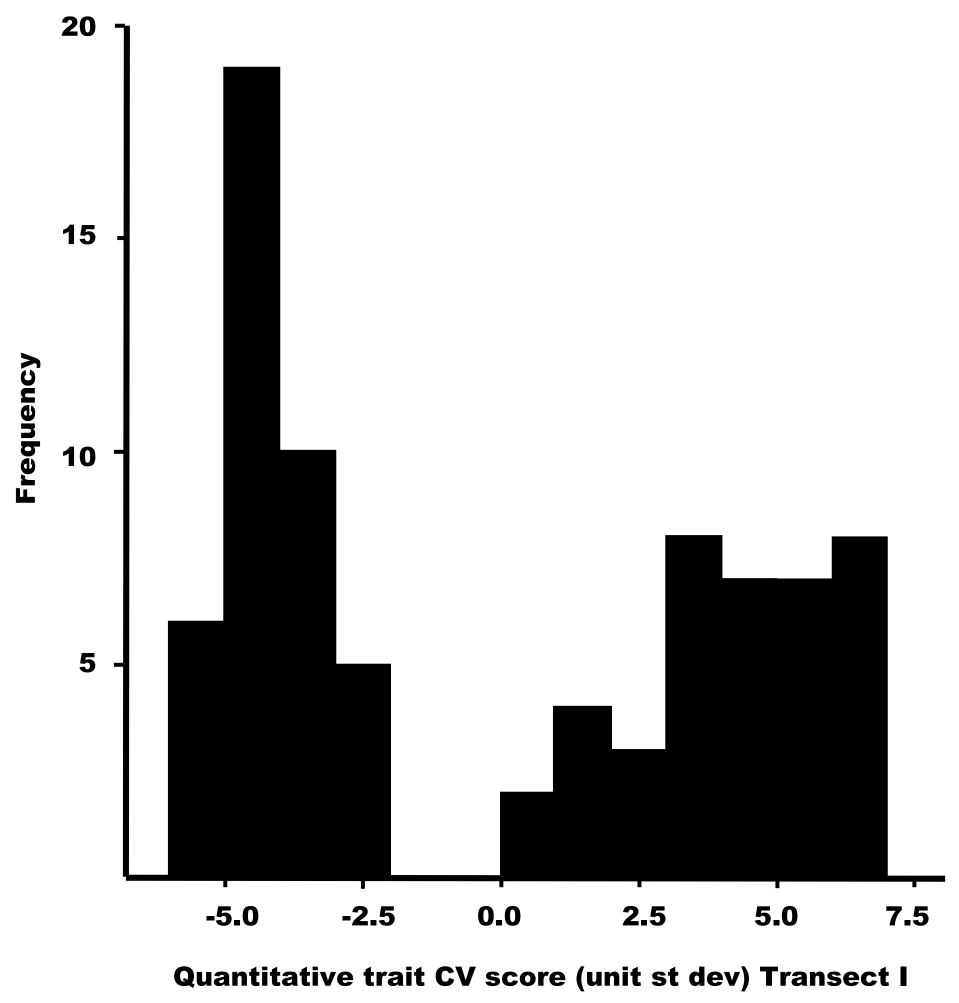

Frequency histogram of individuals along coastal transect I showing bimodality in quantitative traits at this spatial scale. The variable is canonical variate 1 (units in within-group standard deviations). Northwestern precursor individuals are the right mode, central precursor individuals are the left mode, without any overlap.
