# Supplementary material for: Genetic Tests for Ecological and Allopatric Speciation in Anoles on an Island Archipelago
Source: PLoS Genet. 2010 Apr 29;6(4):e1000929. doi: 10.1371/journal.pgen.1000929 (PMC2861690; doi:10.1371/journal.pgen.1000929)
Supplement: Figure S2 — Principal component analyses. Principal component 1 score (vertical axis) for each site (horizontal axis) for transect I = VIII. The legend is as for Figure 3, except r is the correlation between the PC1 score and the frequency of Bayesian clusters when K = 2. (0.03 MB PDF) [file pgen.1000929.s002.pdf]

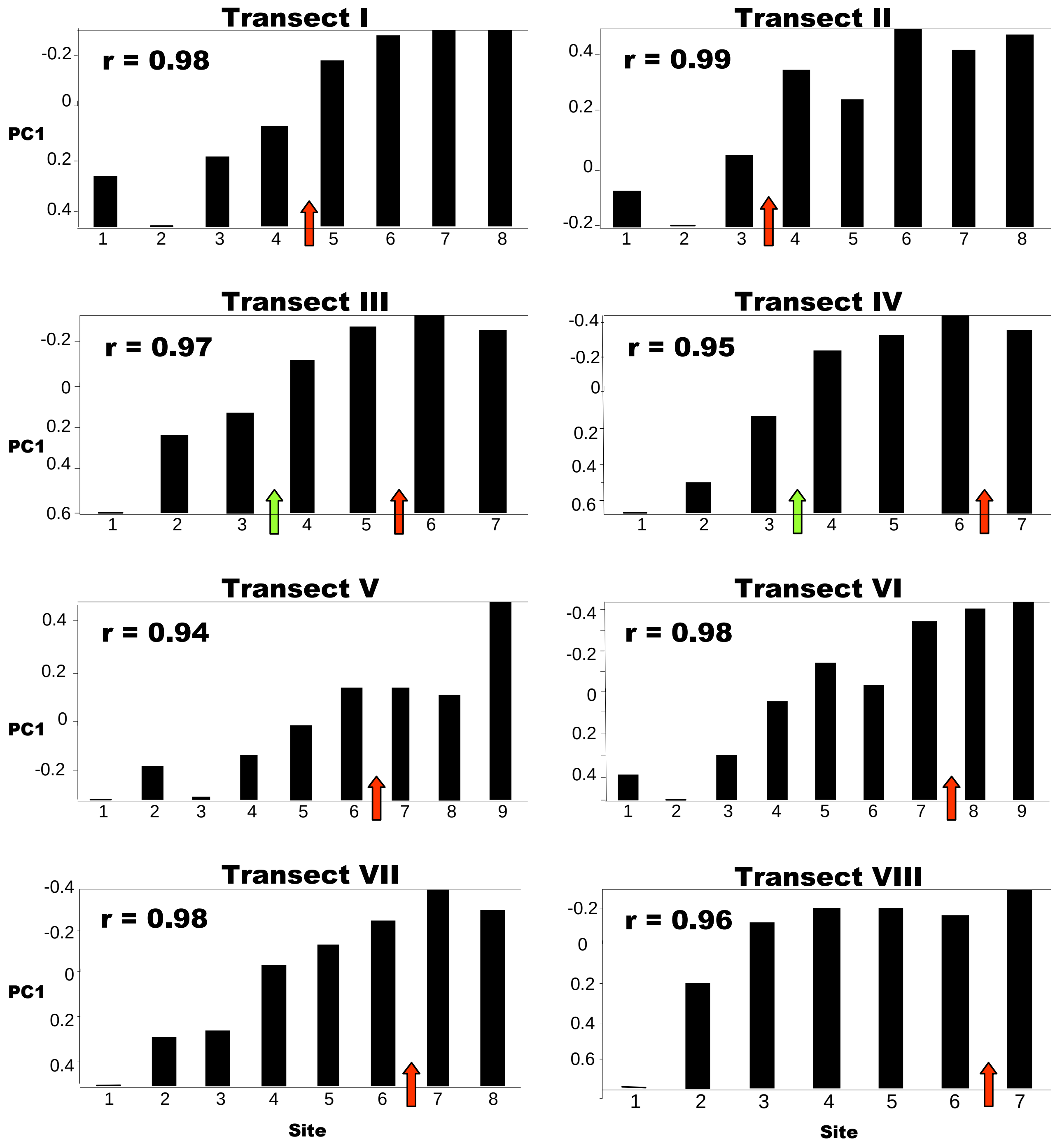

Figure S2. Principal component 1 score (vertical axis) for each site (horizontal axis) for transect I=VIII. The legend is as for Figure 3, except r is the correlation between the PC1 score and the frequency of Bayesian clusters when K=2.
