# Supplementary material for: Genetic Tests for Ecological and Allopatric Speciation in Anoles on an Island Archipelago
Source: PLoS Genet. 2010 Apr 29;6(4):e1000929. doi: 10.1371/journal.pgen.1000929 (PMC2861690; doi:10.1371/journal.pgen.1000929)
Supplement: Table S1 — Mean posterior probabilities (over 10 replicates) for STRUCTURE for K clusters. Maximum values are in bold. (0.01 MB PDF) [file pgen.1000929.s003.pdf]

Table S1. Mean Posterior probabilities (over 10 replicates) for STRUCTURE for K clusters. Maximum values are in bold.

	Transect					
**K**	**I**	**II**	**III-IV**	**V-VI**	**VII-VIII**	**IX**
1	-14055.5	**-13983.9**	-24452.7	-30335.4	-24532.1	**-8714.8**
2	**-13857.5**	-14630.6	**-24239.6**	**-30274.6**	**-24316.9**	-8804.6
3	-13959.5	-14025.1	-24993.1	-30477.9	-24712.1	-8972.6
4	-14078.9	-13987.5	-26166.0	-32765.0	-24610.2	-9235.0
5	-14299.9	-14283.2	-26276.2	-33130.5	-24812.5	-9359.4
6	-17457.1	-20157.6	-26811.0	-32934.3	-25063.4	-9626.7
7	-15284.2	-19871.5	-25623.3	-32970.9	-25660.2	
8	-15714.4	-15749.9	-26451.3	-33316.7	-26510.7	
9	-19867.3	-16193.0	-26483.3	-33810.9	-47350.1	
